# Production performance, egg quality, and uterine gene expression for layers as affected by N-Carbamylglutamate supplementation

**DOI:** 10.3389/fvets.2023.1110801

**Published:** 2023-02-17

**Authors:** Wei Ma, Yi Lu, Chunqiang Wang

**Affiliations:** College of Animal Science and Veterinary Medicine, Jinzhou Medical University, Jinzhou, Liaoning, China

**Keywords:** N-Carbamylglutamate, layer, production performance, egg quality, transcriptome analysis in uterus

## Abstract

**Introduction:**

Supplementation of exogenous additives is a strategy to improve laying performance of layers by regulating uterine function. N-Carbamylglutamate (NCG) as an activator for endogenous arginine synthesis has the potential to regulate the laying performance of layers, but its effects have not been fully understood.

**Methods:**

This study investigated the effects of dietary supplementation of NCG on production performance, egg quality, and uterine gene expression in layers. A total of 360 45-week-old layers with a genetic line of Jinghong No. 1 were used in this study. The experimental period was 14 weeks. All birds were divided into 4 treatments with 6 replicates per treatment and 15 birds per replicate. Dietary treatments were based on a basal diet and supplemented with 0, 0.08, 0.12, or 0.16% NCG to form C, N1, N2, and N3 groups.

**Results and discussion:**

We found that layers in group N1 had higher egg production rate than those in group C. Egg weight was significantly reduced, while eggshell thickness was significantly improved, by treatment. However, the albumen height and Haugh unit were the lowest in group N3. Based on the above results, groups C and N1 were selected for further transcriptomics analysis of uterine tissue by RNA-seq. More than 7.4 Gb clean reads and 19,882 tentative genes were obtained using the *Gallus gallus* genome as a reference. Transcriptomics analysis in uterus tissue revealed that 95 differentially expressed genes (DEGs) were upregulated and 127 DEGs were downregulated. Functional annotation and pathway enrichment analysis showed that DEGs in uterine tissue were mainly enriched in glutathione metabolism, cholesterol metabolism, and glycerolipid metabolism, etc. Vitamin A metabolism-related gene, RBP1, nutrient transport-related gene, ALB, protein synthesis-related gene, METTL21C, and calcium transport-related gene, RYR2, CACNB2, RAMP3, and STAC, were significantly regulated by 0.08% NCG supplementation. Therefore, we concluded that NCG supplementation at a dose of 0.08% improved production performance and egg quality of layers by regulating uterus function.

## Introduction

Egg, as an important source of protein, is widely consumed worldwide. For decades, animal nutritionists have constantly optimized the egg production and egg quality of poultry from the perspective of nutrition to meet people's increasing demand for high-quality eggs (1). High calcium ion transport capacity of the uterus is related to the production of high-quality eggs (2). Huang et al. (3) found that dietary supplementation of mulberry-leaf flavonoids upregulated the expression of calcium ion transport-related genes in uterus, and therefore improved egg quality. Moreover, increasing dietary vitamin A levels was beneficial to ameliorate the reduction of laying performance induced by heat stress (4). Therefore, the reproductive performance of layers is closely related to the supply of exogenous nutrients and the inherent function of ovaries and/or uterus.

N-Carbamylglutamate (NCG) is an activator for endogenous arginine synthesis (5). Its supplementation had positive effects on growth performance and tissue development of broiler chicks (6). Its amnion injection improved the meat quality of broiler chicks (7). Additionally, feeding roosters with NCG-containing diet was beneficial to regulate the levels of circulating reproductive hormone and improve the development of sexual organ (8). For layers, supplementing NCG to the diet can promote the development of follicles (9). Therefore, it seems that supplementing NCG to the diet is beneficial to improve the reproductive performance of birds.

However, nutrients should not only be regarded as a provider of nutrients, but also as a source of various molecules that can be perceived by the organism and affect the expression of the genome (10). Therefore, the application of omics technologies in animal nutrition could identify phenotypic responses of dietary administration of different kinds of additives. Transcriptome sequencing technology (RNA-Seq) can accurately and efficiently obtain almost all the transcripts of a specific tissue in a certain period of time, and deeply excavate the subtle changes in the differential expression of genes in the tissue or cell (11). Therefore, the RNA-Seq technique is of great significance for exploring gene expression and regulation mechanisms at transcription level and is widely used in animal nutrition research (12, 13).

We hypothesized that dietary supplementation of NCG improved production performance and egg quality of layers by regulating the function of uterus. In this study, we first investigated the effects of dietary supplementation of NCG on production performance and egg quality of layer by feeding them with graded levels of NCG-containing diet. Based on the above results, suitable experimental group was selected and then collected uterine tissue for further transcriptomics analysis by RNA-seq. Therefore, the objective of this study was to investigate the effects of dietary supplementation of NCG on production performance, egg quality, and uterine gene expression in layers.

## Materials and methods

All methods and procedures were approved by the Ethics Committee of Jinzhou Medical University and implemented in accordance with the relevant guidelines formulated by the Ministry of Agriculture of the People's Republic of China.

### Experimental design, animals, and housing

A total of 360 45-week-old layers with a genetic line of Jinghong No. 1 were used in this study. All birds were obtained from the poultry experimental unit in Jinzhou Medical University. The experimental period was 14 weeks. All birds were randomly assigned into 4 groups with 6 replicates per group and 15 birds per replicate. Dietary treatments were based on a basal diet and supplemented with 0, 0.08, 0.12, or 0.16% NCG to form C, N1, N2, or N3 groups. The basal diet was (Table 1) formulated according to the recommendation of National Research Council (14). The NCG used in this study was purchased from a commercial company (Anhui Pusheng Pharmaceutical Co. Ltd).

**Table 1:** Composition and nutrient levels of the experimental basal diet (%, as-fed basis).

**Items**	
**Ingredients, %**	
Corn	63.18
Soybean meal	25.60
Dicalcium phosphate	1.30
Limestone	8.70
NaCl	0.26
NaHCO_3_	0.20
Vitamin and mineral premix^a^	0.50
Methionine	0.23
Lysine	0.03
Total	100.00
**Analyzed nutrient composition**	
Metabolizable energy, MJ/kg	11.03
Crude protein, %	16.50
Calcium, %	3.41
Available phosphorus, %	0.32
Lysine, %	0.80
Methionine, %	0.51
Methionine + Cysteine, %	0.80
Tryptophan, %	0.18

^a^Provided per kilogram of diet: 125,000 IU vitamin A; 2,500 IU vitamin D_3_; 10 mg vitamin E; 2 mg vitamin K_3_; 1 mg vitamin B_1_; 5 mg vitamin B_2_; 1 mg vitamin B_6_; 15 mg vitamin B_12_; 500 mg folic acid; 35,000 mg niacin; 10,000 mg Ca-Pantothenate; 50 mg biotin; 8 mg Mn; 60 mg Zn; 25 mg Cu; 40 mg Fe; 0.3 mg Co; 1.5 mg I; 0.15 mg Se.

Layers were housed in natural ventilation and programmable lighting equipped room and reared in an adjacent steel cage, which was installed with nipple drinkers, common trough feed, and egg collection plate. During the experimental period, the average temperature was 23°C. The lighting program was 16 h light and 8 h dark. All layers had free access to feed and water.

### Sampling and measurements

#### Production performance analysis

The number and weight of eggs were recorded daily on a replication basis to calculate egg production rate.

#### Egg quality analysis

On the last day of the experiment, two eggs per replication were selected to measure egg quality. A Vernier caliper was used to measure eggshell thickness excluded inner membrane, which was determined to be based on the average thickness of the rounded end, pointed end, and the middle of the egg. Additionally, the albumen height, Haugh unit, and yolk color were measured using an egg multi-tester (Touhoku Rhythm Co., Ltd., Tokyo, Japan).

#### Transcriptomics analysis of uterus tissue

Based on the results of production performance and egg quality, a total of 6 birds were randomly selected from groups C and N1 for further transcriptomics analysis of uterine tissue by RNA-seq. Birds were euthanized with 1 cc Euthasol^®^ intravenously after 12 h fasting on the last day of the experiment and then remove uterus tissue.

Total RNA was extracted from tissue samples using Trizol reagent. The degree of RNA degradation was analyzed by agarose gel electrophoresis and RNA purity was detected using a Nanodrop 2000 spectrophotometer. The RNA concentration was accurately quantified by Qubit 2.0; and RNA integrity was detected using the Agilent 2100 Bioanalyzer. Following sample testing, a total amount of 3 μg RNA per sample was used as input material for the RNA sample preparations. Sequencing libraries were generated using NEBNext^®^ UltraTM RNA Library Prep Kit for Illumina^®^ (NEB, USA) following manufacturer's recommendations and index codes were added to attribute sequences to each sample (15). The quality of library was assessed on the Agilent Bioanalyzer 2100 system.

The library preparations were sequenced on an Illumina HiSeq. 2500 platform.Quality control of the reads was performed by using in-house written scripts. Raw data of Fastq format were initially processed by in-house perl scripts. In this step, clean reads were obtained by removing reads containing adapter, poly-N, and low-quality reads from raw data. Q20, Q30 and GC content were calculated for the clean data. All downstream analyses were based on clean data with high quality. The PE 150 paired-end sequencing strategy was used in this study. The chicken's genome sequence (90 version) was downloaded from genome website (ftp://ftp.ensembl.org/pub/current_fasta/gallus_gallus/dna/Gallus_gallus.Gallus_gallus-5.0.dna.toplevel.fa.gz). Index of the reference genome was built using Hisat2 v2.0.5 and paired-end clean reads were aligned to the reference genome using Hisat2 v2.0.5. The gene expression level was estimated by the number of normalized fragments per kilogram of transcript per million fragments (FPKM) method. The differential expression analysis of the groups was performed using the DESeq. 2R package (1.16.1) based on the readcount data. The resulting *P* values were adjusted using the Benjamini and Hochberg's approach for controlling the false discovery rate. Genes with an adjusted *P* < 0.05 found by DESeq. Two were assigned as differentially expressed. The group C was used as the control group to analyze the differentially expressed genes in the uterine tissue as affected by NCG supplementation.

Gene ontology (GO) (http://www.geneontology.org/) enrichment analysis of differentially expressed genes was performed using GOseq based on Wallenius non-central hyper-geometric distribution43. This includes three parts: molecular function, biological process, and cellular component. A specific *P*-value was used to determine if a DEG is enriched in the GO. Usually, a *P* < 0.05 is indicative of enrichment. Pathway enrichment analysis was assessed using the KEGG (Kyoto Encyclopedia of Genes and Genomes) database44 (http://www.genome.jp/kegg/). The ClusterProfiler R package was used to test the statistical enrichment of differential expression genes in KEGG pathways. A *P* < 0.05 was considered indicative of the function being enriched.

### Statistical analysis

The normality of data was examined by Shapiro-Wilk test and QQ plots. Then, data were analyzed by one-way ANOVA model with Dunnett's *post hoc* Test using SPSS software (Version 26.0). Results were presented as the means ± standard deviation. Probability value below 0.05 was taken to denote statistical significance.

## Results

Production performance and egg quality as affected by supplementing NCG to the diet of layers was presented in Table 2. We observed that feeding layers with 0.08% NCG-containing diet led to a higher egg production rate in comparison to those fed with a basal diet (*P* < 0.05). Egg weight was affected by treatment, the supplementation of graded levels of NCG resulted in a decrease in egg weight (*P* < 0.05). Additionally, we found that the albumen height (*P* < 0.05) and Haugh unit (*P* < 0.05) were the lowest in N3 group in comparison to those in other groups. Eggshell thickness in N1, N2, and N3 groups was higher than that in C group (*P* < 0.05). However, NCG supplementation had no significant effects on yolk color.

**Table 2:** Effect of dietary supplementation of N-Carbamylglutamate (NCG) on production performance and egg quality in layers.

**Items**	**NCG, %**			
	0.00	0.08	0.12	0.16
Egg production rate, %	86.40 ± 1.67^b^	92.31 ± 5.43^a^	87.01 ± 5.76^b^	89.18 ± 4.12^ab^
Egg weight, g	63.26 ± 1.67^a^	61.33 ± 1.34^b^	61.43 ± 1.56^b^	61.54 ± 1.94^b^
Albumen height, mm	5.84 ± 0.95^a^	6.25 ± 1.01^a^	6.28 ± 0.83^a^	4.46 ± 2.04^b^
Yolk color	4.38 ± 0.74	4.51 ± 1.07	4.75 ± 0.46	5.00 ± 0.93
Haugh unit	73.51 ± 8.19^a^	76.41 ± 6.86^a^	77.13 ± 5.72^a^	53.35 ± 3.23^b^
Eggshell thickness, mm	0.28 ± 0.03^b^	0.31 ± 0.02^a^	0.32 ± 0.03^a^	0.33 ± 0.02^a^

a, b Different superscripts within a column indicate a significant difference (P < 0.05).

As observed in Table 3, after filtering the raw reads, an average of 5,673,995 clean reads were obtained from the samples of group C, whereas an average of 57,340,736 clean reads were obtained from the samples of group N1. Values of Q20 and Q30 were higher than 98% and 94% in both groups, respectively. The contents of GC were higher than 50% for both groups.

**Table 3:** Statistics of sequencing data of uterus in layers.

**Items**	**Clean reads**	**Clean bases**	**Q20, %**	**Q30, %**	**GC content, %**
C_1	55,690,070	8,211,245,214	97.92	93.89	50.51
C_2	50,100,024	7,397,701,348	98.07	94.25	50.23
C_3	64,429,890	9,422,573,881	98.10	94.35	50.49
Average	56,739,995	8,343,840,148	98.03	94.16	50.41
N1_1	58,709,712	8,699,767,426	98.66	96.03	50.40
N1_2	56,509,706	8,375,330,746	98.75	96.33	50.49
N1_3	56,802,790	8,448,341,951	98.61	95.92	50.31
Average	57,340,736	8,507,813,374	98.67	96.09	50.40

**Figure 1:**
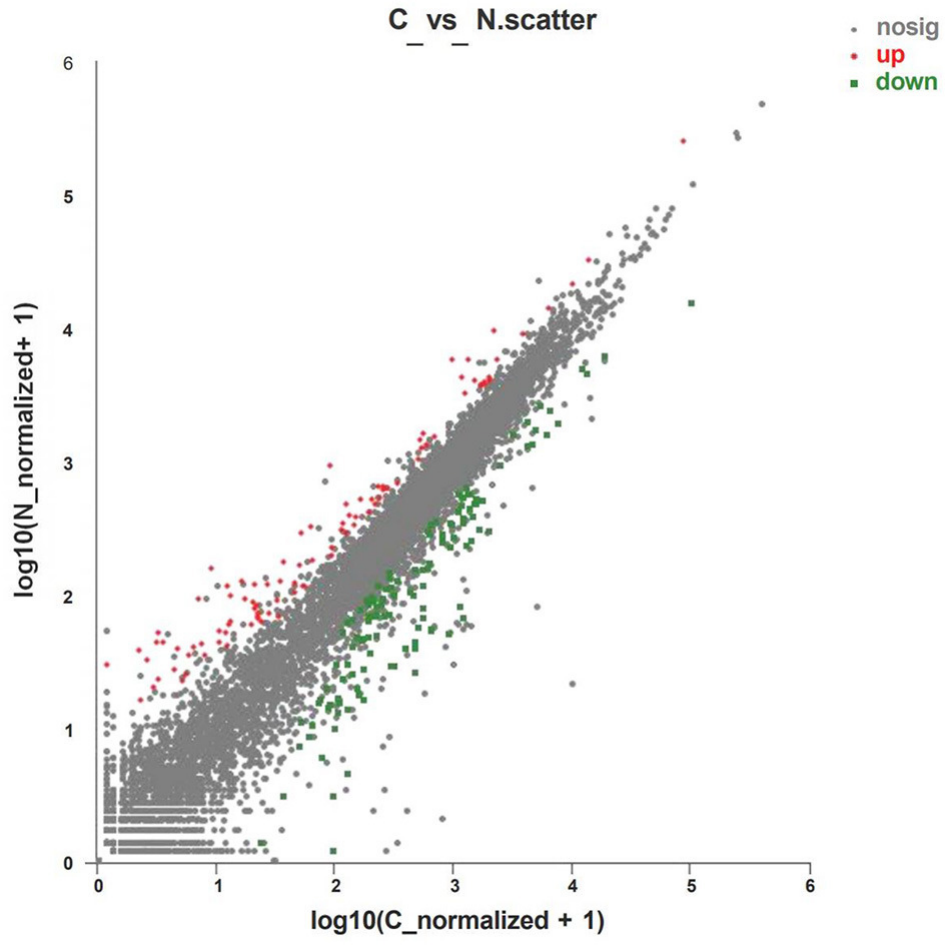
Volcano plot of differentially expressed genes of layers fed with C or N1 groups diets. C, basal diet; N1, basal diet supplemented with 0.08% N-Carbamylglutamate. Nosig, no significant.

Analysis of the expression of differential genes was conducted by using RSEM software. A total of 222 differential genes were obtained (Supplementary material 1), of which 95 genes were significantly upregulated and 127 genes were significantly downregulated (Figure 1).

**Figure 2:**
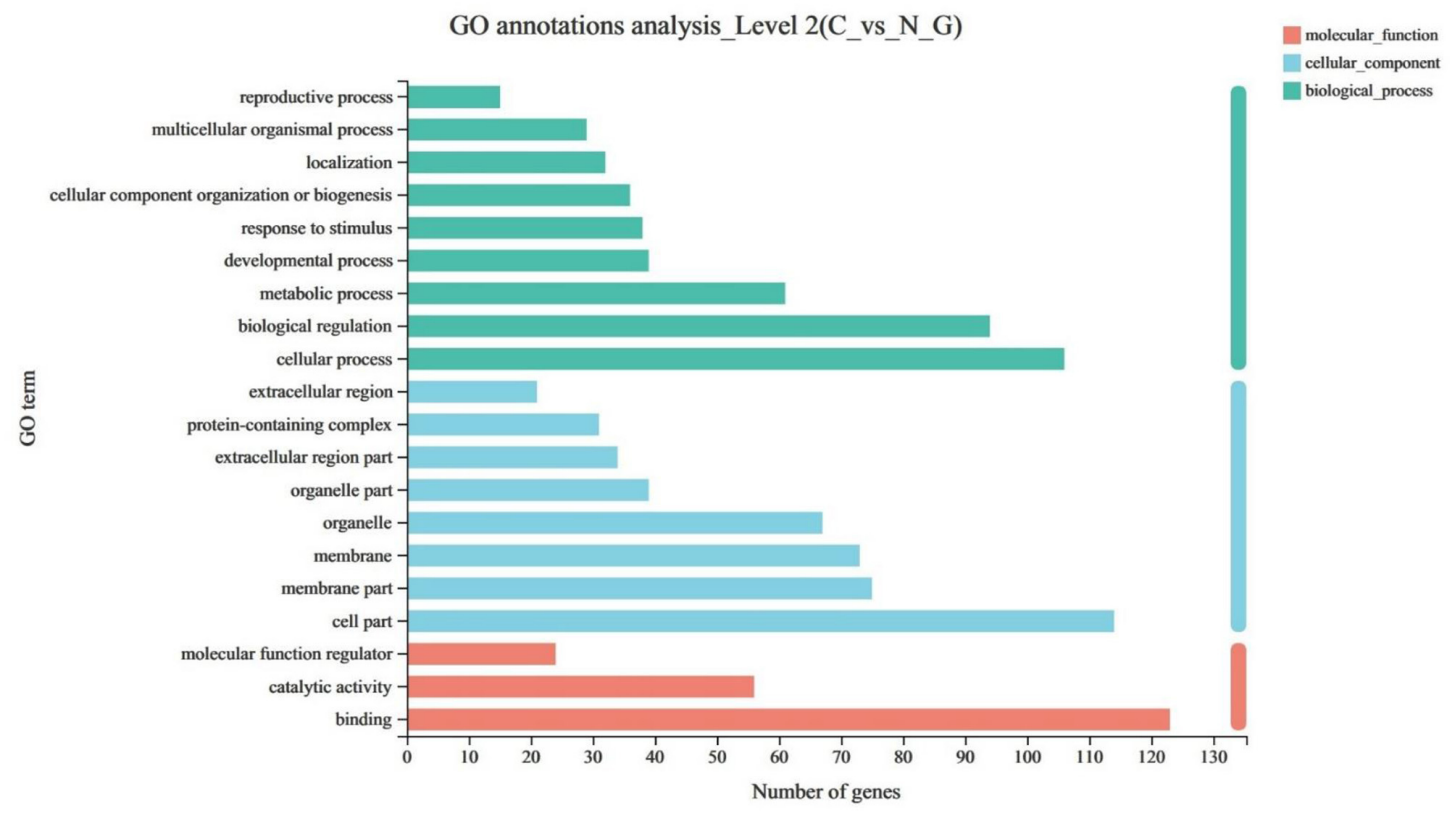
The upregulated items in the GO (gene ontology) database at all levels using DEGs of C vs. N1 groups. C, basal diet; N1, basal diet supplemented with 0.08% N-Carbamylglutamate.

The GO enrichment analysis was used to annotate DEGs and study their distribution to further clarify the function of DEGs. The GO functions were divided into three categories, including biological process, cell component, and molecular function. Among the differential genes, nine differential genes were enriched in biological process, eight differential genes were enriched in cell component, and three differential genes were enriched in molecular function (*P* < 0.05) (Figure 2).

**Figure 3:**
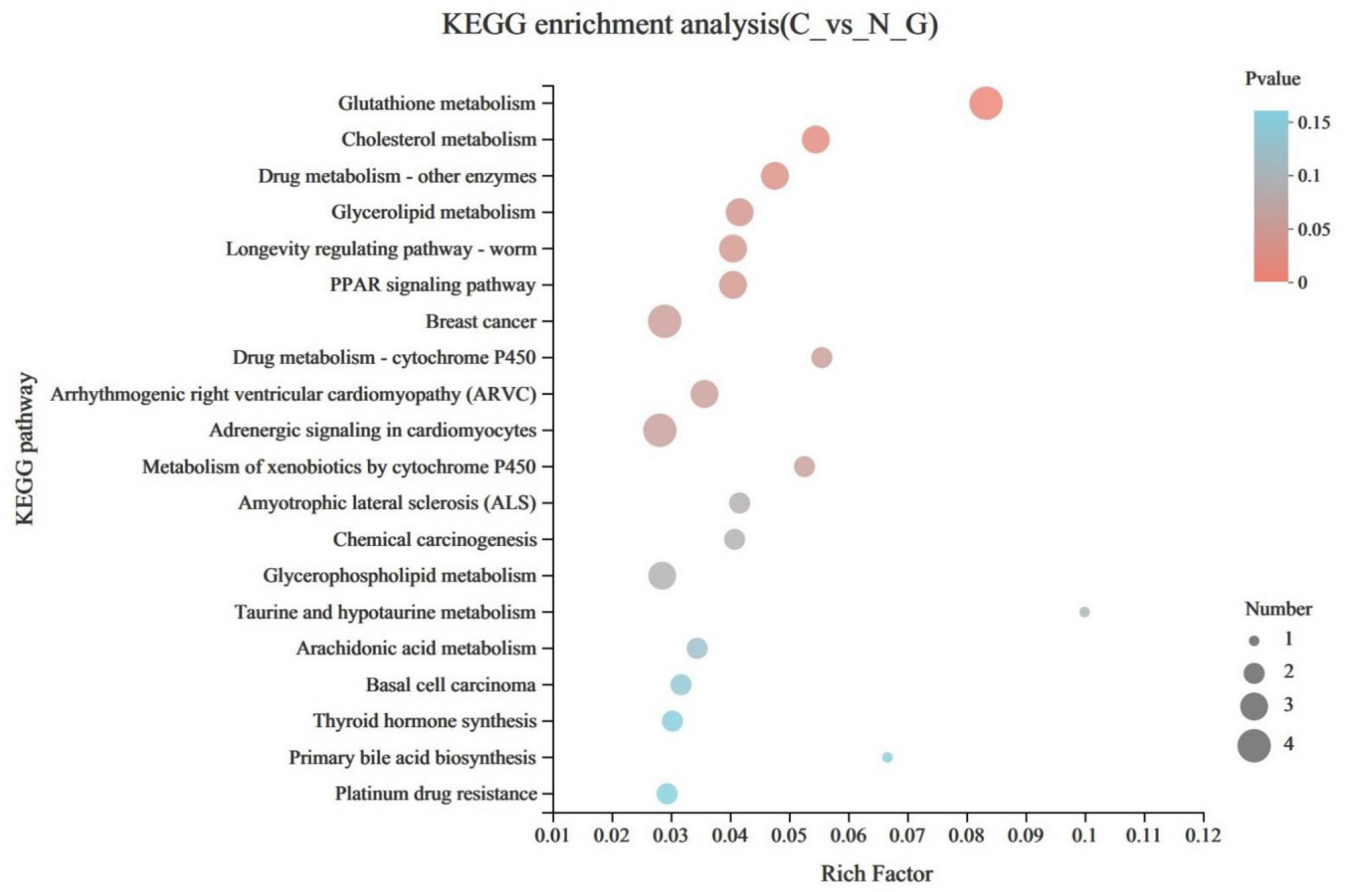
Advanced bubble chart shows significantly enriched pathways based on differentially expressed genes (DEGs) by Kyoto Encyclopedia of Genes and Genomes (KEGG) pathway analysis (*P* < 0.05). The x-axis represents rich factor (rich factor = number of DEGs enriched in the pathway/number of all genes in the background gene set). The y-axis represents the enriched pathway. Color represents enrichment significance, and the size of the bubble represents the number of DEGs enriched in the pathway.

To further identify the major biochemical, metabolic, and signal transduction pathways of the DEGs, we performed a KEGG pathway enrichment analysis. Results indicated that a total of 222 DEGs were enriched in 126 KEGG pathways, of which 11 pathways were significant enriched (*P* < 0.05), mainly involved in glutathione metabolism, cholesterol metabolism, drug metabolism-other enzymes, glycerolipid metabolism, longevity regulating pathway-worm, peroxisome proliferator-activated receptor signaling pathway, breast cancer, drug metabolism-cytochrome P450, arrhythmogenic right ventricular cardiomyopathy, adrenergic signaling in cardiomyocytes, metabolism of xenobiotics by cytochrome P450 (Figure 3).

**Figure 4:**
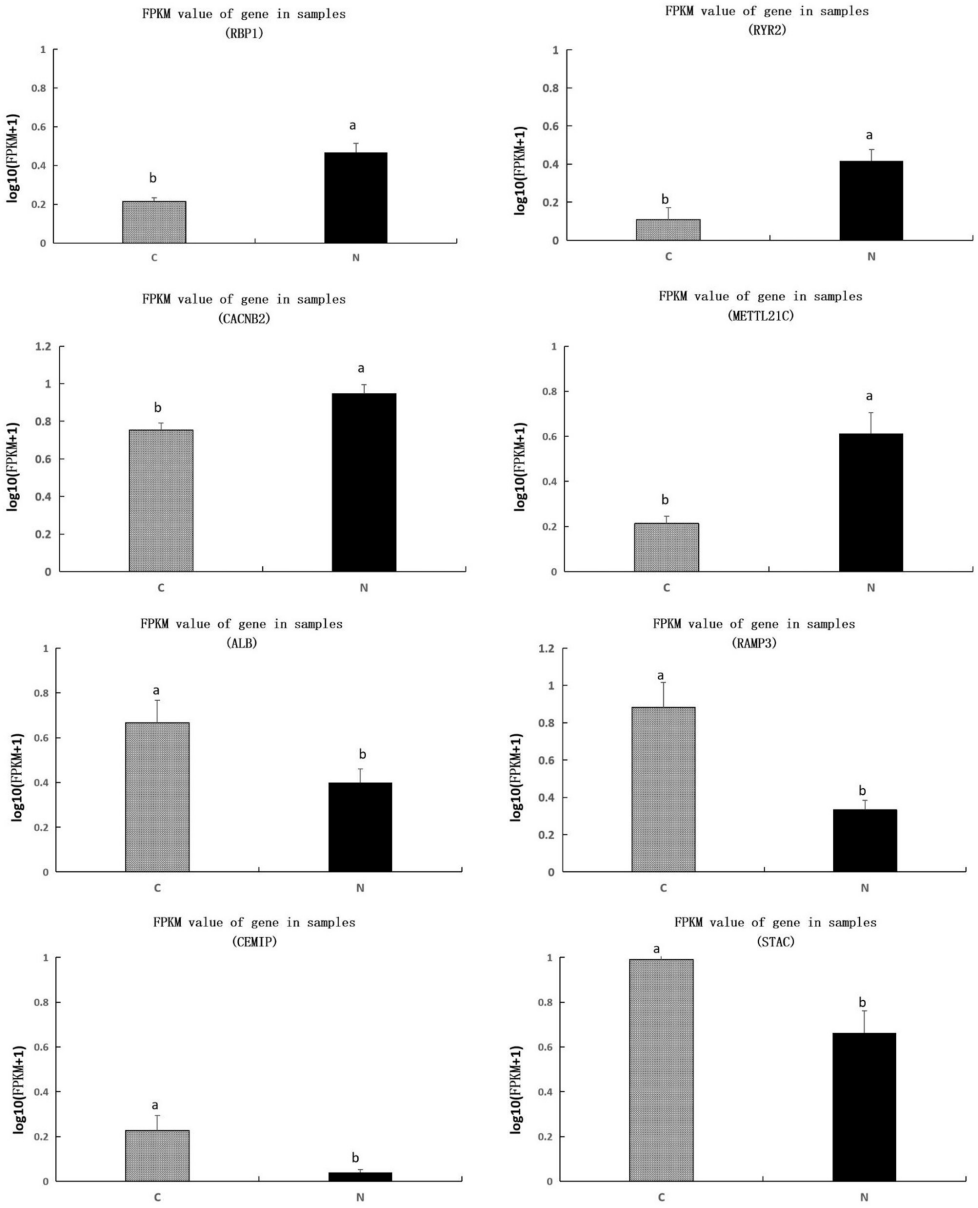
Comparison of transcripts per million values of the RBP1, RYR2, CACNB2, METTL21C, ALB, RAMP3, CEMIP, and STAC genes between C and N1 groups. C, basal diet; N1, basal diet supplemented with 0.08% N-Carbamylglutamate. ^a, b^Means in the same figure with different superscript differ significantly (*P* < 0.05).

We further investigated the value of FPKM for each sample, we found that genes of RBP1, RYR2, CACNB2, METTL21C were significantly upregulated, and genes of ALB, RAMP3, CEMIP, and STAC were significantly downregulated, in group N1 in comparison to those in group C (*P* < 0.05) (Figure 4).

## Discussion

Egg production rate is an important economic parameter for poultry husbandry. In this study, a significant improvement effect on egg production rate was observed when supplementing 0.08% NCG to the diet. The egg production rate of layers is related to the number of preovulatory follicles (16). As reported, Ma et al. (17) noted that NCG supplementation enhanced ovarian angiogenesis and improved follicular development in layers. Additionally, vitamin A is necessary for reproduction. Vitamin A is considered to play a key role in ovarian steroidogenesis, oocyte maturation, and reproductive tract mucous membrane epithelium development (18). Long-term deficiency of vitamin A led to pathological changes in the oviduct mucosa for layers (19). Extra supplementation of vitamin A was beneficial to ameliorate the reduction of laying performance induced by heat stress (4). Vitamin A is transported systemically and intercellularly by RBP. Subsequent transcriptome analysis in this study showed that NCG supplementation at a dose of 0.08% upregulated the expression of RBP1 in uterus. As the action of vitamin A is mediated by RBP. We considered that the dose of NCG at 0.08% seems to be the appropriate level to improve egg production rate, which was partially attributed to the upregulation of RBP1 expression in uterus. However, as the egg production rate of layers is closely related to the number of primordial follicles, further experiments are needed to explore the effects of NCG supplementation on the number of primordial follicles for providing more powerful evidence.

However, we observed that egg weight was negatively affected by treatment. Any dose of NCG used in this study led to a decrease in egg weight. Egg weight is closely related to the quantity of nutrients deposited in eggs (20). ALB gene encodes carrier proteins for a wide range of endogenous molecules (https://www.genecards.org/cgi-bin/carddisp.pl?gene=ALB). Therefore, its expression level in uterus may indicate the situation of nutritional deposition in eggs. In the present study, we observed that NCG supplementation at a dose of 0.08% had downregulation effect on the expression of ALB in uterus. Therefore, a downregulated ALB expression in uterus may be the reason for egg weight loss. The reduction of egg weight has a negative effect on the economic traits of eggs. A low weight of eggs may decrease the acceptability of eggs for consumers.

The Haugh unit is a measure of egg protein quality based on the height of its egg white (albumen) (21, 22). Its measurement determines the protein content and freshness of the egg. Therefore, the results observed in this study indicated that layers fed with 0.16% NCG-containing diet may cause consumers to feel stale. These results also indicated that NCG supplementation may regulate the deposition of protein in eggs. Indeed, as reported, Frank et al. (23) found that oral intake of NCG increased the synthesis of muscle protein in nursing piglets. Zhang et al. (24) reported that NCG supplementation increased nitrogen retention and utilization in Holstein bulls. It is worth noting that the value of albumen height and Haugh unit in group N1 were relatively higher than that in group C, although no statistical difference was observed. The result of transcriptomics analysis indicated that the gene of METTL21C was significantly upregulated by NCG supplementation. METTL21C has been identified as a potential pleiotropic gene for sarcopenia (25). Therefore, the increase of NETTL21C expression in uterus may indicate a high protein synthesis (https://www.informatics.jax.org/marker/MGI:3611450). We considered that the difference between groups C and N1 were masked by the existence of group N3, as the lowest value of albumen height and Haugh unit were presented in group N3. We speculated that the protein synthesis induced by NCG supplementation may have dose-dependent manner. That is, high concentration of NCG has negative effects on protein synthesis, which need to be evaluated in future. In brief, we concluded that feeding layers with 0.16% NCG containing diet had negative effects on albumen height and Haugh unit.

Moreover, we observed that the eggshell thickness was affected by treatment. The supplementation of NCG improved the eggshell thickness. Eggshell is mainly composed of calcium, which is formed in the uterus. The transepithelial transportation of calcium from the blood into the uterus is the predominant route for eggshell calcification (26). Disturbed regulation of calcium transporters expression in uterus contributed to the deterioration of eggshell ultrastructure (27). Wang et al. (28) found that feeding layers with probiotic containing diet increased the expression of calcium transport-related genes, thus improved eggshell quality. In this study, we observed that dietary supplementation of 0.08% NCG upregulated the expression of RYR2 and CACNB2 genes, while downregulated the expression of RAMP3, CEMIP, and STAC genes in uterine tissue. RYR is an intracellular calcium ion release channel in the sarcoplasmic reticulum or the endoplasmic reticulum (29). CACNB2 encodes a subunit of a voltage-dependent calcium ion channel protein that is a member of the voltage-gated calcium channel superfamily (https://www.genecards.org/cgi-bin/carddisp.pl?gene=CACNB2). RAMPS is a type I transmembrane protein with an extracellular N-terminus and a cytoplasmic C-terminus. Its expression leads to the inhibition of bone resorption and enhancement of renal calcium ion excretion (https://www.genecards.org/cgi-bin/carddisp.pl?gene=RAMP3). STAC has been identified as a new regulatory family that plays a role in voltage-gated calcium ion channel transportation (30). Its downregulation induces sperm inactivation by enhancing the concentration of calcium in the semen (31). However, CEMIP involves calcium ion release from the endoplasmic reticulum (32). A downregulated CEMIP expression may indicate the inhibition of calcium release for eggshell formation. But we did not know why NCG supplementation downregulated the expression of CEMIP, an eggshell quality promotion-related gene, which need to be explored in future. However, we can still conclude that the upregulation of RYR2 and CACNB2 was beneficial to increase the calcium ion concentration in uterus, while the downregulation of RAMP3 and STAC was beneficial to release calcium from intracellular storage into uterine environment. Therefore, we considered that the improvement of eggshell thickness caused by supplementing NCG to the diet was related to the upregulation of RYR2 and CACNB2 and the downregulation of RAMP3 and STAC.

In conclusion, we found that feeding layers with 0.08% NCG-containing diet improved egg production rate and egg quality by regulating uterine function. However, the supplementation of NCG had negative effects on egg weight. Additionally, the egg quality was impaired when layers fed with 0.16% NCG-containing diet, which indicated that high-dose of NCG used in layers' diet should be careful. Additionally, we did observe that some genes related to vitamin A metabolism and calcium transport were regulated by NCG supplementation. However, the interesting thing is one of the calcium transport promoting gene downregulated by NCG supplementation. Further study should clarify the role of CEMIP in egg formation.

## Data availability statement

The data presented in the study are deposited in the Figshare repository at the following link: https://figshare.com/articles/dataset/NCG_layer/21992639.

## Ethics statement

Experimental protocol and the process were approved and supervised by the Animal Care and Use Committee of Jinzhou Medical University (Jinzhou, China).

## Author contributions

WM: writing—original draft, investigation, and writing—review and editing. YL: formal analysis and investigation. CW: conceptualization, methodology, supervision, and writing—review and editing. All authors contributed to the article and approved the submitted version.
